# PARP1 and XRCC1 exhibit a reciprocal relationship in genotoxic stress response

**DOI:** 10.1007/s10565-022-09739-9

**Published:** 2022-07-01

**Authors:** Julia M. Reber, Jovana Božić-Petković, Michelle Lippmann, Marvin Mazzardo, Asisa Dilger, Rebecca Warmers, Alexander Bürkle, Aswin Mangerich

**Affiliations:** Molecular Toxicology Group, Department of Biology, University of Konstanz, 78457 Constance, Germany

**Keywords:** PARP1, ARTD, Poly(ADP-ribosyl)ation, XRCC1, Camptothecin

## Abstract

PARP1 (aka ARTD1) acts as a prime sensor of cellular genotoxic stress response. PARP1 detects DNA strand breaks and subsequently catalyzes the formation of poly(ADP-ribose) (PAR), which leads to the recruitment of the scaffold protein XRCC1 during base excision and single strand break repair and the assembly of multi-protein complexes to promote DNA repair. Here, we reveal that the recruitment of either protein to sites of DNA damage is impeded in the absence of the other, indicating a strong reciprocal relationship between the two DNA repair factors during genotoxic stress response. We further analyzed several cellular and molecular endpoints in HeLa *PARP1* KO, *XRCC1* KO, and *PARP1*/*XRCC1* double KO (DKO) cells after genotoxic treatments, i.e., PARylation response, NAD^+^ levels, clonogenic survival, cell cycle progression, cell death, and DNA repair. The analysis of NAD^+^ levels and cytotoxicity after treatment with the topoisomerase I inhibitor camptothecin revealed a hypersensitivity phenotype of *XRCC1* KO cells compared to *PARP1* KO cells—an effect that could be rescued by the additional genetic deletion of *PARP1* as well as by pharmacological PARP inhibition. Moreover, impaired repair of hydrogen peroxide and CPT-induced DNA damage in *XRCC1* KO cells could be partially rescued by additional deletion of *PARP1*. Our results therefore highlight important reciprocal regulatory functions of XRCC1 and PARP1 during genotoxic stress response.

**Supplementary Information:**

The online version contains supplementary material available at 10.1007/s10565-022-09739-9.

## Introduction

Poly(ADP-ribosyl)ation (PARylation) is an essential posttranslational modification (PTM) that plays key roles in in cellular stress responses towards a wide spectrum of genotoxicants, such as DNA oxidating, alkylating, and crosslinking agents, as well as UV and ionizing radiation (Martin-Hernandez et al. 2017; Ray Chaudhuri and Nussenzweig 2017). Specifically, PARylation plays an active role in several DNA repair pathways, such as base excision repair (BER) and single strand break repair (SSBR), nucleotide excision repair (NER), and DNA double-strand break repair (DSBR) (Azarm and Smith 2020; Eisemann and Pascal 2020), during DNA replication (Hanzlikova and Caldecott 2019a), and by regulating different forms of cell death (Aredia and Scovassi 2014b). The modification is mediated by some members of the family of “ADP-ribosyl transferases diphtheria toxin-like” (ARTDs) (also known as PARPs) and requires NAD^+^ as a substrate to generate the biopolymer poly(ADP-ribose) (PAR) (Luscher et al. 2021). By cleaving the glycosidic bond, nicotinamide is released and ADP-ribose moieties are covalently attached at several different amino acid residues of hundreds of target proteins (Leidecker et al. 2016; Leslie Pedrioli et al. 2018; Martello et al. 2016; Zhang et al. 2013). PARP1 as the most prominent member of the PARP family is predominantly activated by DNA single or double strand breaks, which are directly induced by DNA damaging agents or which occur as DNA repair intermediates in response to genotoxic stress (Horvath et al. 2011; Rank et al. 2016). PAR chains can vary considerably in chain length and structure, extending up to 200 ADP-ribose units in length and adopting either linear or branched conformations (Alemasova and Lavrik 2019; Reber and Mangerich 2021). Apart from covalent attachment to a wide range of target molecules, PAR can also non-covalently interact with target proteins via diverse PAR-binding modules (Reber and Mangerich 2021; Teloni and Altmeyer 2016). Due to the action of PAR-catabolizing enzymes, PARylation is a fully reversible PTM (O'Sullivan et al. 2019; Rack et al. 2021). Thereby, during genotoxic stress response, PARPs and PARylation modulate the properties, activities, and the intracellular localization of their target proteins and influence the dynamics of assembly and disassembly of protein complexes in a spatio-temporal manner. Furthermore, PARylation affects cellular (patho-)physiology by its interference with NAD^+^ metabolism (Azarm and Smith 2020; Eisemann and Pascal 2020; Martin-Hernandez et al. 2017; Ray Chaudhuri and Nussenzweig 2017).

Due to their prominent role in DNA repair and the DNA damage response, PARPs have emerged as auspicious targets in cancer therapy. This is highlighted by the clinical use of PARP inhibitors (PARPi) either as sensitizers in combination with chemotherapy or as stand-alone drugs against the backdrop of synthetic lethality (Curtin and Szabo 2020; Lord and Ashworth 2017). While the application of PARPi in tumors deficient in BRCA-dependent homologous recombination is the most common example of this concept, additional approaches have increasingly been explored in recent years (Pilie et al. 2019). As a result, synthetic lethality could be shown for PARPi in combination with other genetic constellations in specific tumors, including XRCC1-deficient sporadic cancers (Ali et al. 2018, 2020). The molecular scaffold protein XRCC1 is an essential factor within the BER and SSBR, representing a central platform for the coordination of various other DNA repair enzymes like polynucleotide kinase 3′-phosphatase (PNKP), DNA polymerase β, and DNA ligase 3 (Caldecott 2019; Polo et al. 2019). It is closely connected to PARP1 (Caldecott et al. 1996; Masson et al. 1998) and able to non-covalently interact with PAR chains via a conserved PAR binding motif comprised of basic and hydrophobic amino acids and/or a phosphate-binding pocket within its central BRCT1 domain (Breslin et al. 2015; Li et al. 2013; Pleschke et al. 2000). PARP1 activity is important for XRCC1 function and its recruitment to sites of DNA damage and stalled replication forks (Campalans et al. 2013; El-Khamisy et al. 2003; Okano et al. 2003; Ronson et al. 2018)(Ying et al. 2016). Recently, we showed that the recruitment of XRCC1 to DNA damage sites further depends on PAR chain length, as expression of PARP1 variants producing short polymer led to reduced recruitment of XRCC1 to sites of laser-induced DNA damage (Aberle et al. 2020). Additionally, PARylation has been shown to be a key factor in the regulation of XRCC1 chromatin binding and in its nucleoplasmic retention, preventing it from re-entering nucleoli after the induction of oxidative stress (Hanzlikova et al. 2017a; Veith et al. 2019). Conversely, XRCC1 seems to be important for the regulation of PAR-mediated caspase-independent cell death (Keil et al. 2006). Interestingly, hereditary mutations in XRCC1 can result in neurological disorders in humans and mice. Of note, in *Xrcc1*-deficient mouse models, these pathologies could be reduced or prevented by deletion or pharmacological inhibition of PARP1 (Hoch et al. 2017; Komulainen et al. 2021). With respect to a possible molecular mechanism of those findings, the Caldecott group reported that XRCC1 prevents endogenous PARP1 trapping during BER in RPE-1 cells. Thus, in the absence of XRCC1, PARP1 trapping at sites of DNA damage impaired BER and increased sensitivity towards genotoxic treatment with methyl-methane sulfonate (MMS). Conversely, in the absence of PARP1, XRCC1 appears to be dispensable for BER under the conditions tested (Demin et al. 2021). In a follow-up study, the group showed that the XRCC1 deficient cells exhibit reduced potential to recover from transcriptional blockage following H_2_O_2_-induced DNA base damage, which appeared to be caused by excessive PARP1 activity (Adamowicz et al. 2021).

Apart from classical BER and SSBR, PARP1, and XRCC1 play a crucial role in the genotoxic response to camptothecin (CPT)-mediated topoisomerase I (Top1) poisoning, and, consequently, combinations of PARP and topoisomerase 1 inhibitors have been tested in clinical cancer therapy (Chowdhuri and Das 2021; Mei et al. 2020; Thomas and Pommier 2019). Mechanistically, CPT traps the Top1-DNA cleavage complex (Top1cc), which abolishes the DNA religation step and directly leads to DNA single strand breaks and protein-DNA crosslinks. The resulting 5′-hydroxyl DNA end can be detected by PARP1, which leads to the recruitment of XRCC1, tyrosylphosphodiesterase 1 (TDP1), and PNKP, and processing of the damage for further repair via the subsequent BER machinery (Das et al. 2014; Mei et al. 2020). In addition to this immediate DNA damaging effect of CPT treatment, during S-phase persistent Top1cc may convert to replication fork run-offs and toxic DNA double-strand breaks (DSBs), when encountered by the replication machinery (Mei et al. 2020). In this case, PARP1 contributes to replication fork reversal, which may contribute to the religation of Top1cc (Ray Chaudhuri et al. 2012). It is noteworthy that while the repair of directly induced CPT damage relies on both PARP1 and XRCC1, CPT-induced replication stress reversal also involves PARP1, yet XRCC1 presumably only plays a minor role in the repair of this kind of damage (Mei et al. 2020; Thomas and Pommier 2019; Ying et al. 2016).

In the present study, we further analyzed the complex interplay between PARP1 and XRCC1 during genotoxic stress responses. To this end, we analyzed HeLa *PARP1* knockout (*P1* KO) (Rank et al. 2016), *XRCC1* knockout (*X1* KO), and *PARP1/XRCC1* double-knockout (*P1/X1* DKO) cell lines (Aberle et al. 2020) with regard to their responses to different genotoxic treatments, namely H_2_O_2_ and camptothecin (CPT) for a comprehensive spectrum of cellular endpoints such as PARylation response, NAD^+^ levels, clonogenic survival, cell cycle progression, cell death, and DNA repair. Furthermore, to investigate the reciprocal relationship between PARP1 and XRCC1 within the DNA damage response, we reconstituted HeLa *PARP1/XRCC1* DKO cells with fluorescently labelled versions of these proteins and analyzed recruitment kinetics after the site-specific induction of DNA strand breaks via laser-irradiation.

## Material and methods

### Western blot analysis of protein expression

Cells were seeded in 6-well plates, harvested with trypsin–EDTA (Gibco), and resuspended in PBS after pelleting. SDS loading dye [93.75 mM Tris–HCl (pH 6.8), 9 M urea, 7.5% (v/v) β-mercaptoethanol, 15% (v/v) glycerol, 3% (w/v) SDS, 0.01% (w/v) bromphenol blue] was added to cells (at 1 × 10^6^ cells per 100 µL), then samples were heated at 95 °C for 5 min. Subsequently, 20 µL of each sample (corresponding to equivalents of 2 × 10^5^ cells) were loaded onto a 10% SDS gel. Western blot analysis was performed using a 0.45 µm nitrocellulose membrane (GE Healthcare) and TBSM-T [5% (w/v) non-fat milk, 150 mM NaCl, 10 mM Tris base pH 8, 0.05% Tween20] as blocking solution. The membrane was then incubated with the following primary antibodies in TBSM-T overnight at 4 °C: mouse-anti-PARP1 (CII10, purified from culture supernatant of hybridoma cells, 1:300), rabbit-anti-XRCC1 (Enzo Life Sciences, 1:1000) and mouse-anti-β-actin (Cell Signaling, 1:1000). Respective secondary antibodies were incubated in TBSM-T for 1 h at room temperature (RT): goat-anti-mouse HRP-coupled or goat-anti-rabbit HRP-coupled (both Dako, 1:5000). Following, the chemiluminescent signals were detected on an ImageQuant LAS4000 imager (Cytiva).

### Analysis of cell proliferation

Cell lines were seeded in triplicates in five 96-well flat-bottom plates (t_0_, t_1_, t_2_, t_3_ and t_4_). After approx. 4 h, when cells had sufficient time to attach to the well, 10 µL Alamar blue dye (ThermoFisher Scientific) was added to each well of one 96-well plate (t_0_). Fluorescence was measured after 4 h (λ_ex_ 560 nm, λ_em_ 595 nm) on an Infinite F200 pro plate reader (Tecan). The procedure was repeated every 24 h with another well plate (t_1_ – t_4_). Obtained values were first normalized to the medium control (containing only cell culture medium and Alamar blue dye) and then to the respective values at time point t_0_.

### Analysis of PAR formation

Cells were seeded in 12-well plates on glass cover slips. After 24 h, PAR formation was induced by treatment with 500 µM H_2_O_2_ for 7.5, 15, or 30 min and cells were subsequently washed. All washing steps were performed in PBS. Cells were fixed for 20 min using 4% (w/v) PFA in PBS, the reaction was stopped by adding 100 mM glycine in PBS for 1 min followed by a washing step. Cells were then permeabilized for 3 min in 0.4% TritonX-100 in PBS, followed by another washing step and blocking in PBSM-T [5% (w/v) non-fat milk, 0.5% Tween20, in PBS]. In between the following steps, samples were always washed three times for 10 min each. Then, cells were incubated with the primary antibody mouse-anti-PAR (10H, purified from culture supernatant of hybridoma cells, 1:300 in PBSM-T) overnight at 4 °C. The respective secondary antibody goat-anti-mouse IgG coupled to Alexa546 (Invitrogen, 1:300 in PBSM-T) was incubated for 1 h at RT. DNA was stained with 0.2 µg/mL Hoechst33342 in PBS for 5 min and cover slips were subsequently mounted on microscopy slides using Aqua Poly/Mount (Polysciences). Microscopic images were acquired using a Zeiss AxioObserver microscope. Image data was analyzed with regards to PAR intensity using an automated KNIME workflow as described previously (Rank et al. 2016).

### *NAD*^+^*cycling assay*

Cells were seeded in 6-well plates. The next day, cells were treated with 500 µM H_2_O_2_ for 10 min or 10 µM CPT for 1 h (1% DMSO as a solvent control). Where applicable, cells were pre-treated with 10 µM veliparib or 10 µM olaparib (both Selleck Chemicals) for 30 min. After the indicated periods, cells were harvested using trypsin–EDTA, pelleted and resuspended in 500 µL ice-cold PBS. Afterwards, cell numbers were determined, and cells were lysed by addition of 24 µL 3.5 M perchloric acid for 15 min. Samples were then centrifuged and supernatant was transferred to pre-cooled tubes. Precipitation was achieved by addition of 350 µL ice-cold phosphate buffer [0.33 mM K_2_HPO_4_, 0.33 mM K_2_PO_4_ in H_2_O, pH 7.5]. Immediately after, samples were frozen in liquid nitrogen and stored at − 80 °C until measurement. For analysis, samples were thawed, centrifuged and supernatant was transferred to pre-cooled tubes. From there, 40 µL of each sample were added to a 96-well flat bottom plate in triplicates and diluted with 160 µL diluent [0.5 M H_3_PO_4_, 0.5 M NaOH in H_2_O, pH 10.5]. As a standard, NAD^+^ (Sigma-Aldrich) was diluted in diluent to concentrations ranging from 0.01 µM to 0.5 µM. Finally, all standards and samples were incubated with 100 µL reaction mix [0.48 M bicine (pH 8), 4 mg/mL BSA, 0.02 M EDTA, 2.4 M ethanol, 2 mM MTT, 3 µM alcohol dehydrogenase, 5.7 mM phenazine ethosulfate] for 30 min at 30 °C. Subsequently, absorption was measured at 550 nm, with a reference wavelength of 690 nm, on an Infinite F200 pro plate reader (Tecan). NAD^+^ levels were calculated from a corresponding NAD^+^ standard curve and values were normalized to the total cell number of the respective sample.

### Annexin V/PI cell viability analysis

Cell lines were seeded in 6-well plates. The next day, cells were treated with 1, 10, or 100 µM CPT or 1% DMSO as a solvent control. Where applicable, treatment solution contained 10 µM veliparib or 10 µM olaparib. After 42 h, cells were harvested using trypsin–EDTA, washed in ice-cold PBS and resuspended in Annexin binding buffer [ABB; 10 mM HEPES pH 7.4, 140 mM NaCl, 2.5 mM CaCl_2_] to a concentration of 1 × 10^6^ cells per mL. A volume of 195 µL cell suspension was added to 5 µL Annexin V-APC (BioLegend; 50 ng/µL in ABB) and incubated for 15 min at RT in the dark. Subsequently, 200 µL propidium iodide (PI) solution (Sigma-Aldrich; 1 µg/mL in ABB) were added and samples were analyzed using a LSRFortessa flow cytometer (BD).

### Cell cycle analysis

Cells were seeded in 6-well plates. The next day, cells were treated with 5, 10, or 25 nM CPT or 1% DMSO as a solvent control. After 42 h, cells were harvested using trypsin–EDTA, pelleted and resuspended in 300 µL ice-cold PBS to a concentration of 3.3 × 10^6^ cells per mL. After addition of 700 µL ice-cold ethanol, cells were incubated for 1 h on ice, washed twice in PBS and then resuspended in 300 µl PBS. Subsequently, 200 µL DNA staining buffer [5 µg/mL PI solution, 5 µg/mL RNase, in PBS] were added, samples were incubated for 30 min at RT in the dark and analyzed using a LSRFortessa flow cytometer (BD).

### Colony formation assay

Cells of the different cell lines were seeded in 12-well plates. The next day, cells were treated with CPT in concentrations as indicated for 24 h or 1% DMSO as a solvent control. After treatment, cells were harvested using trypsin–EDTA, the number of viable cells was determined, and 500 cells were plated in 6-well plates in technical triplicates. After 10 days, colonies were washed with PBS, fixed with 10% formaldehyde and stained with 0.1% crystal violet. The number of colonies was determined by using OpenCFU software (Geissmann 2013). The surviving fraction was calculated by dividing the average of treated samples by the average of untreated controls.

### FADU assay

Cells were seeded in 96-well round-bottom culture plates. The next day, cells were treated with 100 µM H_2_O_2_ for 30 min or 1 µM CPT for 60 min. Subsequently, treatment medium was exchanged for fresh cell culture medium and cells were either incubated for 60 or 240 min to allow time for DNA repair or directly used for further analysis. Therefore, cells were washed in 100 µL ice-cold PBS and the DNA integrity was determined via fluorometric detection of alkaline DNA unwinding (FADU). The following pipetting steps were automated by a liquid handling device. First, 7.5 µL PBS and 7.5 µL lysis buffer [400 mM guanidine thiocyanate, 30 mM EDTA, 0.2% N-lauroylsarcosyl pH 12], both supplemented with 300 µM DFA and 300 µM TEMPOL, were added to each well and plates were incubated for 12 min at 4 °C. After cell lysis, 270 µL dilution buffer [8.4 mM HEPES] was added, followed by incubation for 1 h at 37 °C. ‘T-value samples’ (T values reflect the amount of total DNA) were then treated with 150 µL neutralization buffer [1 M D-( +)-glucose monohydrate, 14 mM β-mercaptoethanol], before all samples were treated with 50 µL alkaline unwinding buffer [200 mM NaOH in 40% lysis buffer] and incubated for 1 h at 30 °C to allow for DNA unwinding. Following a cool-down to 22 °C for 30 min, ‘P-value samples’ (P-values serve as a reciprocal measure for cellular DNA strand breaks) were then also treated with 150 µL neutralization buffer. For fluorescence measurement, 50 µL SYBRGreen I solution (ThermoFisher Scientific; 1:8.333 in ddH_2_O) were added to each well and the signal intensity was determined (λ_ex_ 485 nm, λ_em_ 535 nm) using an Infinite F200 plate reader (Tecan). The amount of double-stranded DNA compared to the whole DNA was calculated by dividing the mean of P-values by the mean of corresponding T-values (both measured in technical quadruplicates) (Mack et al. 2021; Moreno-Villanueva et al. 2009).

### Recruitment of PARP1 and XRCC1 to sites of laser-induced DNA damage

*PARP1/XRCC1* DKO cells were seeded in µ-dishes (Ibidi). After 18 h, cells were transfected with expression constructs encoding either eGFP-N1::PARP1 or mRFP-C1::XRCC1 or with both constructs in parallel. For microscopic analysis, cell culture medium was replaced by phenolred-free medium 24 to 40 h after transfection. A Zeiss LSM700 inverted laser-scanning microscope, equipped with a multi-color femtosecond fiber laser setup was employed for microirradiation and following time-lapse imaging (Schmalz et al. 2018). In situ bandwidth-limited optical pulses (center wavelength 1035 nm) were focused onto the sample with a 63 × oil immersion objective lens (N.A. 1.4), using an average optical power between 16 and 18 mW. The laser was scanned along a 4.6 µm linear track (52-pixel positions, pixel dwell time 45 ms). In each experiment, at least 27 cells were irradiated in three sets of 9 and the experiments were performed at least three times on different days. Quantitative image analysis was performed in FIJI with the BIC Macro Toolkit (BIC toolbox, University of Konstanz, Germany; available for download at https://www.biologie.uni-konstanz.de/bioimaging-centre/). To ensure comparability between different experiments, only cells with similar nuclear fluorescence intensity values were included in the evaluation). [N.B. Results of cells reconstituted with both, XRCC1 and PARP1, are based on a previous data set published in (Aberle et al. 2020) using adapted evaluation parameters. Data of single-transfected cells (i.e., either XRCC1 or PARP1) were acquired in parallel experiments with identical microscopic settings and are published here for the first time.]

## Results

### Comparative characterization of HeLa PARP1 knockout, XRCC1 knockout,and PARP1/XRCC1 double-knockout cells

**Fig. 1:**
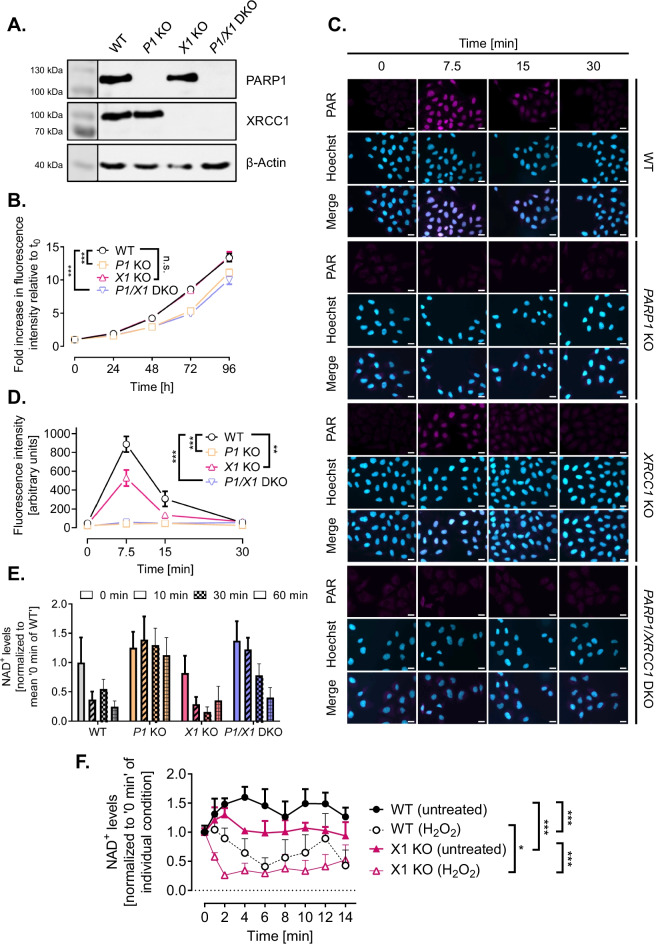
Cellular characterization of HeLa WT, *PARP1* KO (*P1* KO), *XRCC1* KO (*X1* KO), and *PARP1/XRCC1* double KO (*P1/X1* DKO) cell lines. **A** Western blot analysis of PARP1 and XRCC1 expression in HeLa WT and KO cells. β-Actin served as a loading control. **B** Analysis of cell proliferation via Alamar Blue assay. Means ± SEM of *n* = 4–5 independent experiments. Statistical analysis was performed using two-way ANOVA testing. **C** Single-cell immuno-epifluorescence analysis of PAR formation after treatment with 500 μM H_2_O_2_ for the indicated times, using the PAR-specific antibody 10H. Representative images of *n* = 3 independent experiments. Scale bars indicate 20 µm. **D** Densitometric quantification of epifluorescence imaging data of PAR signals as shown in (**C**) using an automated KNIME workflow. Means ± SEM of *n* = 3 independent experiments. Statistical analysis was performed using two-way ANOVA testing. **E** Analysis of NAD^+^ levels using an enzymatic NAD^+^ cycling assay after treatment with 500 μM H_2_O_2_ for time points as indicated. Means ± SEM of *n* = 4 independent experiments. **F** Time-course analysis of NAD.^+^ levels after treatment with 500 µM H_2_O_2_. Values were normalized to respective untreated controls. Means ± SEM of *n* = 3 independent experiments. Statistical analysis was performed using two-way ANOVA testing. *KO* knockout, *DKO* double knockout

We previously generated genetic *PARP1* and *XRCC1* single as well as double knockout HeLa cell lines using TALEN and CRISPR/Cas9 technologies (Aberle et al. 2020; Rank et al. 2016). For the current study, we first validated that all used cell lines display a complete abrogation of the respective protein expression via Western blot analysis (Fig. 1A) As it is evident from Fig. 1A, the genetic knock-out of PARP1 or XRCC1 had no obvious influence on the expression levels of the respective other protein thereby verifying previous results (Aberle et al. 2020). To analyze how the loss of either PARP1 or XRCC1 or both proteins together affect basic cellular phenotypic parameters, we analyzed cell proliferation via an Alamar Blue assay. While *XRCC1* KO cells proliferated at the same rate as HeLa WT cells, proliferation was significantly slower for *PARP1* KO and *PARP1/XRCC1* DKO cells, with differences already detectable after 48 h (Fig. 1B). To analyze how XRCC1 abrogation potentially affects the PARylation response upon genotoxic stress, we examined PAR levels after treatment with 500 µM H_2_O_2_ via immunofluorescence staining, using the PAR-specific antibody 10H. Over the course of 30 min, we observed similar PAR formation and degradation dynamics for HeLa WT and *XRCC1* KO cells, with a peak at 5–10 min and a return to basal levels after approximately 30 min (Fig. 1C, D). [*N.B*., The H_2_O_2_ treatment concentration and time points of analysis were chosen based on data from previous studies (Aberle et al. 2020; Rank et al. 2016).] The maximum fluorescence intensity was, however, reduced in *XRCC1* KO cells to about 60% compared to WT cells, indicating lower levels of 10H-detectable PAR formation in the absence of XRCC1. In accordance with PARP1 producing the bulk of cellular PAR, *PARP1* KO and *PARP1/XRCC1* DKO cells showed no or very low signal intensities, signifying the absence of a (strong) PARylation response. These data are supported by the analysis of intracellular NAD^+^ levels 10 min after treatment with 500 µM H_2_O_2_. NAD^+^ levels remained steady 10 min after H_2_O_2_ treatment for *PARP1* KO and *PARP1/XRCC1* DKO cells, yet a trend for decreased NAD^+^ was evident in HeLa WT and *XRCC1* KO cells at 10 min and later time points (Fig. 1E). While no drop in NAD^+^ levels was observed in PARP1 KO cells until 60 min post treatment, NAD^+^ levels started to decline in *PARP1/XRCC1* DKO cells at 30 min and 60 min posttreatment, in a PARP1-independent manner. The NAD^+^ decline in WT and *XRCC1* KO cells was confirmed in time course analyses up to 14 min, which revealed a significantly faster drop in NAD^+^ levels after H_2_O_2_ treatment in *XRCC1* KO cells compared to WT cells, leading to massive depletion of NAD^+^ already by 2 min posttreatment in *XRCC1* KO cells compared to 6 min in WT cells (Fig. 1F).

In summary, the genetically engineered HeLa cells used in this study display a complete genetic knockout of *PARP1*, *XRCC1*, or both, respectively, and show gene constellation-specific phenotypes with regards to cell proliferation as well as PARylation and NAD^+^ metabolism in response to genotoxic stress induced by H_2_O_2_ treatment.

### Recruitment of PARP1 and XRCC1 to sites of laser-induced DNA damage

**Fig. 2:**
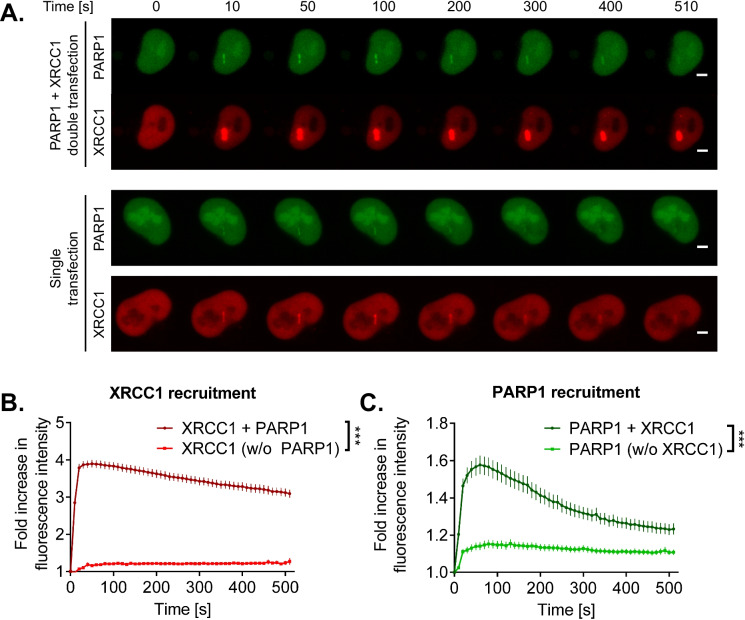
Recruitment and dissociation kinetics of XRCC1 and/or PARP1 at sites of laser-induced DNA damage in HeLa *PARP1/XRCC1* DKO cells. **A** Representative microscopic images. Scale bar indicates 5 μm. **B**, **C** Quantification of mRFP-XRCC1 and/or PARP1-eGFP recruitment dynamics from image data as shown in **A**. Means ± SEM of ≥ 109 cells (PARP1) or ≥ 97 cells (XRCC1) in *n* = 4 independent experiments. Statistical analysis was performed using two-way ANOVA testing. **B** Recruitment of mRFP-XRCC1 either alone (w/o PARP1), or in combination with PARP1-eGFP (“ + PARP1”). **C** Recruitment of PARP1-eGFP either alone (w/o XRCC1), or in combination with mRFP-XRCC1 (“ + XRCC1”). N.B., results of double-transfected cells reconstituted with both, XRCC1 and PARP1, are based on a data set previously published in (Aberle et al. 2020) with adapted evaluation parameters. Data of single transfected cells (either XRCC1 or PARP1) were acquired in parallel experiments with identical microscopic settings and are published here for the first time

As PARP1 and XRCC1 are essential factors within the DNA damage response and their functions have been shown to be tightly connected, we analyzed the recruitment of the two proteins to site-specific, laser-induced DNA damage. We therefore reconstituted *PARP1/XRCC1* DKO cells with fluorescently tagged versions of PARP1 and/or XRCC1. In cells reconstituted with both proteins, XRCC1 showed a fast recruitment with maximum signal intensities at the irradiated site after 50 s, followed by a slow dissociation over 500 s [N.B., results of control experiments with PARP1/XRCC1 double transfected cells are based on a previously published data set (Aberle et al. 2020)]. In the absence of PARP1 on the other hand, as expected, recruitment of XRCC1 barely exceeded background levels and reached a low-level steady state after approximately 100 s (Fig. 2A, B). In turn, PARP1 likewise showed a relatively fast recruitment in the presence of XRCC1, with maximum signal intensities 60 s after induction of DNA damage. Subsequently, we observed a dissociation from the damage site and a substantial release 500 s post irradiation (Fig. 2A, C). Interestingly, in the absence of XRCC1, PARP1 recruitment to sites of DNA damage was strongly impeded both with regards to velocity and maximum signal intensities (Fig. 2C). While peak recruitment was reached only 80–130 s postirradiation; thereafter, a steady-state low-level accumulation at DNA damage sites was observed for the following 400 s, without significant release from the irradiation site. For comparison, in *PARP1* KO cells, which expressed endogenous XRCC1, reconstituted PARP1-eGFP showed a very similar recruitment behavior as it was observed in double reconstituted *PARP1/XRCC1* DKO cells (Aberle et al. 2020), further supporting the specificity of the results of the present study.

In summary, these results confirm that recruitment of XRCC1 to sites of DNA damage is dependent on the presence of PARP1, but recruitment of PARP1, in turn, appears to be dependent on the presence of XRCC1 as well, highlighting a strong reciprocal relationship between the two factors. The reduced recruitment of PARP1 in the absence of XRCC1 may also relate to the reduced PAR formation after H_2_O_2_ treatment, as shown in Fig. 1C, D.

### Impact of PARP1 and XRCC1 deletion on the cellular response after camptothecin treatment

**Fig. 3:**
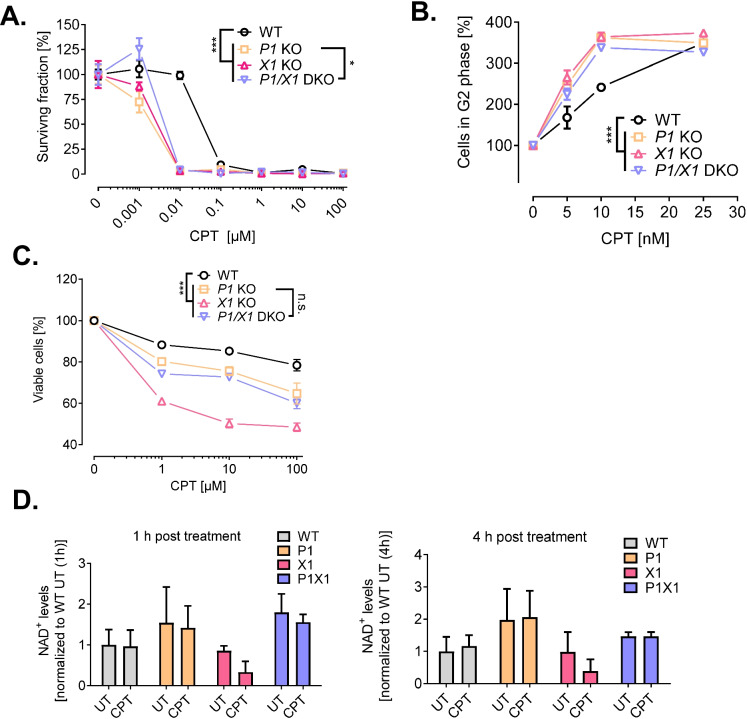
Response of HeLa WT, *PARP1* KO (P1 KO), *XRCC1* KO (X1 KO), and *PARP1/XRCC1* DKO (P1/X1 DKO) cells to camptothecin (CPT) treatment. **A** Clonogenic survival assay of cells treated with CPT at concentrations as indicated for 24 h and afterwards incubated for 10 days prior to colony evaluation. Means ± SEM of *n* = 3 independent experiments. Data of the individual cell lines are normalized to the respective untreated solvent control. **B** Cell cycle analysis via PI staining and subsequent flow cytometric analysis. Cells in G2 phase relative to solvent control after CPT treatment as indicated. Means ± SEM of *n* = 3–5 independent experiments (*n* = 1 for treatment with 25 nM CPT)*.* The full dataset including data on cell fractions in G1 and S phase is shown in Suppl. Figure 1. **C** Cytotoxicity analysis via annexin V/PI staining and subsequent flow cytometric analysis. Cells were treated with CPT concentrations as indicated for 42 h. Means ± SEM of *n* = 3 independent experiments. The full data set on annexinV/PI positive cells is shown in Suppl. Figure 2. **D** NAD^+^ levels after treatment with 10 μM CPT for 1 h and 4 h, as measured by an enzymatic NAD.^+^ cycling assay. Means ± SEM of *n* = 3 independent experiments. For all data sets, statistical analyses were performed using two-way ANOVA testing

The results on the apparent reciprocal relationship between PARP1 and XRCC1 within the DNA damage response prompted us to further analyze the interplay between the two proteins at various levels of cellular physiology. To this end, we treated cells with CPT, which is known to induce DNA single strand breaks and DNA–protein adducts as well as DSBs as a consequence of collapsed replication forks in S phase (Mei et al. 2020; Thomas and Pommier 2019). As mentioned above, it has been demonstrated that both, PARP1 and XRCC1, are involved in the repair of CPT induced SSB, while PARP1, but not XRCC1, is involved in the resolution of CPT-induced replication stress and DNA double strand breaks. We analyzed clonogenic survival, cell cycle progression, cytotoxicity, and cellular NAD^+^ levels to obtain a comprehensive picture of the cellular stress response in the context of the specific genetic constellations (Fig. 3). As it is evident from Fig. 3A, *PARP1* and *XRCC1* KO sensitized cells to CPT treatment with regards to clonogenic survival without revealing major differences between the different genetic constellations—yet demonstrating the epistatic contribution of PARP1 and XRCC1 in the clonogenic survival of HeLa cells after CPT treatment. Further expanding on those findings, we treated the different cell lines with sublethal concentrations of CPT in the low nanomolar range and analyzed cell cycle status (Fig. 3B and Suppl. Fig. S1). While all four genotypes showed a concentration-dependent increase in fractions of the G2 phase, HeLa WT cells were—in full concordance with the results obtained from the clonogenic survival analysis—less sensitive to the treatment with CPT at concentrations < 10 nM. While such a low-dose treatment with CPT as conducted in colony formation and cell cycle analysis leads to the induction of replication stress and DNA double-strand breaks, CPT treatment at higher doses induces a significant amount of direct DNA single strand breaks (Mei et al. 2020; Thomas and Pommier 2019). Therefore, to test the acute cytotoxic response of CPT, we treated cells in concentrations in the micromolar range for 42 h and performed annexin V/PI flow cytometric analysis. In untreated cells, we observed that none of the KO cell lines displayed a reduced basal viability compared to HeLa WT cells. Furthermore, we observed increased cytotoxicity after CPT treatment in a concentration-dependent manner for all four cell lines (Fig. 3C and Suppl. Figure 2A). However, in case of such micromolar CPT concentrations, differences in sensitivity towards CPT treatment were revealed between the specific genotypes. Thus, while HeLa WT cells showed to be least sensitive, *XRCC1* KO cells were most strongly sensitized already at 1 µM CPT, the lowest concentration tested in this experimental setup. Interestingly, cells missing both PARP1 and XRCC1 were less sensitive than the cells missing XRCC1 only, matching the sensitivity of *PARP1* KO cells. These results suggest that XRCC1 is strongly involved in the response mechanisms of HeLa cells towards micromolar CPT treatment and that the sensitivity of *XRCC1* KO cells can be rescued by an additional deletion of PARP1 (Fig. 3C). With regards to the type of cell death, most of the dead cells were annexin V + PI positive, followed by PI positive cells, with only a minority of cells being detected to be solely annexin V positive, without revealing any significant differences in the specific type of cell death between CPT concentrations and genotypes (Suppl. Fig. S2A). These data suggest that the major route of cell death upon micromolar CPT treatment is via necrotic mechanisms. To test if cytotoxicity is a consequence of PARP1 overactivation and associated NAD^+^ depletion, we analyzed NAD^+^ levels 1 h and 4 h upon CPT treatment (Fig. 3D). Interestingly, upon CPT treatment, NAD^+^ levels dropped only in *XRCC1* KO cells at both time points, which could be rescued by additional genetic depletion of *PARP1*, This finding conceivably represents a connection to the increased sensitivity in CPT-induced cytotoxicity observed for the *XRCC1* KO cells in comparison to *PARP1*/*XRCC1* KO cells.

In summary, analysis of the genetic interplay of *PARP1* and *XRCC1* upon CPT treatment revealed a general hypersensitivity of PARP1 and/or XRCC1 deleted cells with regard to colony formation, cell cycle progression, and acute cytotoxicity. In case of micromolar CPT treatment, gene constellation specific differences were revealed pointing to a rescue effect of *XRCC1* KO hypersensitivity by an additional *PARP1* KO, which is potentially related to the inhibition of a drop in intracellular NAD^+^ levels.

**Fig. 4:**
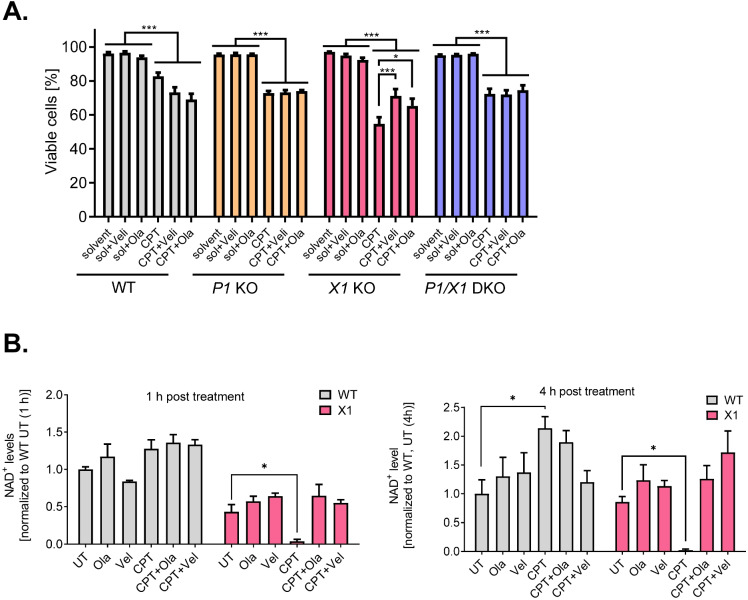
PARP inhibitor treatment partially rescues sensitivity of *XRCC1* KO cells towards CPT toxicity. **A** Viable cells as analyzed by Annexin V/PI flow cytometry after treatment with 10 μM CPT for 42 h ± 10 μM PARP inhibitor veliparib (Veli) or olaparib (Ola). Full data set including individual fractions of annexin V/PI positive cells is shown in Suppl. Figure 2B. Statistical analysis was performed using two-way ANOVA testing. **B** NAD^+^ levels after treatment with 10 μM CPT for 1 h and 4 h ± 10 μM PARP inhibitor Veli or Ola, as measured by an enzymatic NAD.^+^ cycling assay. Means ± SEM of *n* = 3 independent experiments. Statistical analysis was performed using one-way ANOVA testing

Having observed the rescue effect of the *XRCC1* KO phenotype by additional abrogation of *PARP1* on a genetic level, we next analyzed if such a rescue effect can also be replicated by means of pharmacological PARP inhibition. To this end, we treated cells with 10 µM CPT and additionally with 10 µM PARP inhibitor (PARPi) and subsequently analyzed cell viability via Annexin V/PI flow cytometric analysis. While the PARPi treatment sensitized HeLa WT cells to CPT, reducing their viability from over 80% to approximately 70%, *PARP1* KO and *PARP1/XRCC1* DKO cells were unaffected by PARPi treatment, as expected. For *XRCC1* KO cells, which again displayed the strongest sensitivity towards CPT treatment, both veliparib and olaparib were able to partially rescue the viability from approximately 55 to 71% and 65%, respectively (Fig. 4A and Suppl. Figure 2B), thus mimicking the effect observed with *PARP1/XRCC1* DKO cells (Fig. 3C). Next, we analyzed NAD^+^ levels in WT and *XRCC1* KO cells with or without PARPi treatment to examine if the PARPi-dependent partial rescue of the CPT-induced cytotoxicity in *XRCC1* KO cells is also related to a protection of NAD^+^ depletion. Indeed, both veliparib and olaparib treatment prevented NAD^+^ depletion after CPT treatment for 1 and 4 h, leading to levels similar to those of *XRCC1* KO cells exposed to solvent control (Fig. 4B).

In summary, these experiments demonstrate that *PARP1* KO, *XRCC1* KO and *PARP1/XRCC1 DKO* cells are sensitive to CPT treatment with regard to clonogenic survival, G2 arrest, and acute cytotoxicity. Moreover, in micromolar concentrations, *XRCC1* KO cells are even more sensitive to CPT treatment compared to *PARP1* KO cells. Interestingly, this hypersensitivity effect could be rescued in *PARP1/XRCC1 DKO* cells as well as by treatment with the PARPi olaparib and veliparib. Furthermore, the observed rescue effect is reflected in intracellular NAD^+^ levels, as depletion of NAD^+^ levels was rescued in *PARP1/XRCC1* DKO cells as well as by PARPi treatment.

### Impact of PARP1 and/or XRCC1 deletion on DNA strand break induction and repair

**Fig. 5:**
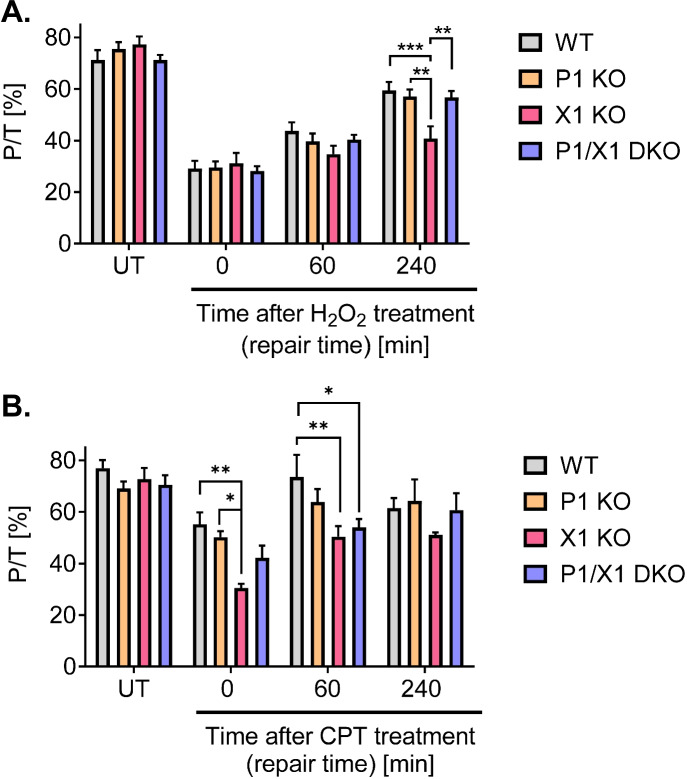
Automated FADU analysis of DNA strand break induction and repair after DNA damaging treatment of HeLa WT, *PARP1* KO (P1 KO), *XRCC1* KO (X1 KO), and *PARP1/XRCC1* DKO (P1/X1 DKO) cells. P/T-values provide a measure for the amount of cellular DNA strand breaks. P-values are derived from SYBR Green fluorescence of samples that have undergone alkaline unwinding, whereas corresponding T-values are derived from SYBR Green fluorescence of samples without alkaline unwinding and serve as a normalization control for the total amount of DNA per sample. **A** Cells were either untreated (UT) or treated with 100 μM H_2_O_2_ for 30 min and were afterwards incubated for times as indicated to allow DNA repair. Means ± SEM of *n* = 8–9 independent experiments. **B** Cells were either untreated (UT) or treated with 1 µM CPT for 60 min and were afterwards incubated for times as indicated to allow DNA repair. Means ± SEM of *n* = 5 independent experiments. Statistical analysis was performed using two-way ANOVA testing

Next, we investigated how the different genotypes affect DNA strand break induction and repair after treatment with H_2_O_2_ and CPT by using an automated version of the fluorometric detection of alkaline DNA unwinding (FADU) assay (Fig. 5). In this 96-well plate assay, the obtained readout are ratios of P to T values (P/T), which represent a measure for DNA integrity, i.e., the lower the P/T values the more strand breaks are present in cellular DNA (Mack et al. 2021; Moreno-Villanueva et al. 2009). P-values are derived from SYBR Green I fluorescence intercalated into double stranded DNA of samples undergone alkaline unwinding treatment, whereas corresponding T-values are derived from SYBR Green I fluorescence of samples without alkaline unwinding and serve as a normalization control for the total amount of DNA per sample (*c.f.*, Material and Methods). Figure 5A shows that while the P/T value was at about 75% for untreated cells of all genotypes, it was strongly reduced to approximately 30% for all cell lines after treatment with 100 µM H_2_O_2_ for 30 min, indicating the effective induction of DNA strand breaks under these treatment conditions. Over the course of 240 min after treatment, HeLa WT, *PARP1* KO, and *PARP1/XRCC1* DKO cells were able to efficiently repair the damage and P/T-values increased to around 60%. In contrast, *XRCC1* KO cells were impaired in DNA repair, since in this case the P/T value increased only by 10% over the 4-h repair period. For cells treated for 1 h with 1 µM CPT, immediately after CPT treatment, the strongest DNA strand break induction was observed for *XRCC1* KO cells with a P/T value of 30.5%. In comparison, WT cells showed levels of 55%, *PARP1* KO of 50%, and *PARP1/XRCC1* of 42.2%, indicating a partial rescue of the XRCC1 hypersensitivity (Fig. 5B). As with H_2_O_2_ treatment, repair of DNA strand breaks was observed 60 min and 240 min after the removal of CPT, with a tendency towards slower repair in *XRCC1* KO cells in comparison to the other genotypes.

In summary, automated FADU experiments revealed a defect of *XRCC1* KO cells in the repair H_2_O_2_-induced DNA damage, which could be rescued by additional *PARP1* deletion, analogous to the rescue phenotype in *XRCC1/PARP1* DKO and PARPi-treated *XRCC1* KO cells observed for CPT-induced cytotoxicity.

## Discussion

PARP1 and XRCC1 represent key proteins intimately involved in BER, SSBR, and CPT-induced DNA damage repair with distinct relationships that are not fully understood yet (Caldecott 2019; Chowdhuri and Das 2021; Demin et al. 2021). For example, it is well known that recruitment and retention of XRCC1 at specific sites of DNA damage is dependent on PARP1 and PAR [e.g., (Aberle et al. 2020; Breslin et al. 2015; Kim et al. 2015; Veith et al. 2019)]. Furthermore, as key repair factors, both proteins are relevant players in cancer therapy. While PARP1 inhibition, on the one hand, has been shown effective in inducing synthetic lethality in XRCC1-deficient sporadic cancers (Ali et al. 2018, 2020), XRCC1 protein levels, on the other hand, demonstrate correlation with cancer resistance to CPT treatment, which is reversible by PARP inhibition (Park et al. 2002). In terms of underlying molecular mechanisms, two recent studies from the Caldecott group revealed that XRCC1 can prevent PARP1 trapping at DNA damage, otherwise resulting in continuous PARP1 activation during BER with implications in transcriptional recovery upon oxidative stress treatment (Adamowicz et al. 2021; Demin et al. 2021). These results highlight that PARP1 and XRCC1 are mechanistically tightly interlinked during BER/SSBR, and that the relationship is presumably not unidirectional, but rather reciprocal. In the present study, we used previously generated *PARP1* and *XRCC1* single- and double-knockout HeLa cell lines (Aberle et al. 2020; Rank et al. 2016) to comprehensively analyze their genetic interaction with respect to a number of phenotypic and mechanistic parameters, such as cell proliferation, PARylation and NAD^+^ metabolism, recruitment behavior to sites of laser-induced DNA damage, different forms of genotoxic stress response, and DNA repair.

Reflecting the role of PARP1 during DNA replication and cell cycle (Hanzlikova and Caldecott 2019b; Slade 2019), *PARP1* KO and *PARP1/XRCC1* DKO cells showed reduced proliferation rates even under non-stressed conditions, while *XRCC1* KO cells proliferated under non-stress conditions as WT cells did (Fig. 1). Furthermore, our results confirmed that *PARP1* KO and *PARP1/XRCC1* DKO cells display strongly reduced levels of PAR formation, and consequently no measurable decline in NAD^+^ levels upon H_2_O_2_ treatment. This is in accordance with previous data, showing that PARP1 is responsible for the bulk of genotoxic stress-induced PAR formation in HeLa cells (Horvath et al. 2011; Rank et al. 2016). In contrast, lowered NAD^+^ levels were detected in HeLa WT and *XRCC1* KO cells after CPT treatment, indicative of CPT-induced activation of PARP1 in *XRCC1*-deficient cells. Interestingly, peak PARylation levels after H_2_O_2_ treatment were reduced in *XRCC1* KO cells compared to HeLa WT cells. In comparison, Demin et al. observed an increased PARylation response in RPE-1 *XRCC1* KO cells at 15 min post-MMS treatment, which declined below the levels reached in WT cells at 60 min post treatment. Results from both studies can be reconciled when considering the technical conditions and specific reagents employed in the individual experiments. While immunofluorescence analysis of the present study was performed with the PAR specific monoclonal 10H antibody, which preferentially recognizes longer PAR chains > 10 ADP-ribose units, Demin et al. used a commercial anti-PAR binding reagent, which also detects oligo-ADP-ribosylated proteins. Data of the present study and by Demin et al. (2021) are therefore in accordance with the conclusion that upon genotoxic treatment, PAR molecules in *XRCC1* KO cells exhibit reduced chain length and branching complexity. Demin et al. suggested that although PARP1 is initially engaged excessively with BER intermediates and is initially hyperactive in *XRCC1* KO cells during MMS treatment, it becomes progressively less active and unable to dissociate from BER intermediates thereafter. They further assumed that progressive PARP1 inactivation during BER in *XRCC1* KO cells can be a result of NAD^+^ depletion, which is in line with our data showing an immediate drop in NAD^+^ levels (Fig. 1). Similarly, in XRCC1-deficient CHO EM9 cells a stronger depletion of NAD(P)H could be shown after treatment with MMS, as compared to EM9 cells expressing human XRCC1. Application of the PARP inhibitors 3-AB and DPQ in turn rescued the MMS-induced effect (Nakamura et al. 2003). The same effect of NAD(P)H reduction and rescue by 3-AB was also described in MMS-treated human breast cancer cell lines MDA-MB-453 and MDA-MB-549 (Brem and Hall 2005). In the present study, potent PARP inhibitors veliparib and olaparib were able to diminish the CPT-induced effect, rescuing NAD^+^ levels in addition to improving viability of *XRCC1* KO cells (Fig. 4). Taken together, we propose that the cytotoxic effects of CPT, especially on *XRCC1* KO cells, are at least partly caused by PARP1-dependent NAD^+^ exhaustion, which is absent in the *PARP1/XRCC1* DKO cell line and which can be prevented by pharmacological PARP inhibition. In this regard it will be interesting to test if also elevated NAD^+^ levels, e.g., by supplementation of NAD^+^ precursor molecules, can have similar effects.

Demin et al. further demonstrated that PARP1 accumulates in chromatin in *XRCC1* KO cells upon treatment with MMS. They concluded that elevated levels of SSBs in *XRCC1* KO cells upon MMS treatment were a consequence of PARP1 trapping and that XRCC1 acts as an anti-trapper of PARP1. Their data suggest that Polβ and Lig3, which are recruited by XRCC1 to sites of DNA damage, actively suppress PARP1 activation at SSBs created during BER and that the presence of XRCC1 greatly promotes this suppression. If this happens because of direct molecular interactions of the XRCC1/Polβ/Lig3 complex with PARP1 to repel PARP1 at the damage site or if PARP1 is released as a secondary effect, due to efficient completion of lesion repair and therefore disappearance of PARP1’s DNA binding target, remains a question to be clarified. In accordance with these results, our data from laser-micro irradiation experiments revealed reduced dissociation kinetics of PARP1 from sites of laser-induced DNA damage in the absence of XRCC1. Remarkably, those micro-irradiation experiments also revealed that the peak levels of PARP1 molecules recruited to sites of laser-induced DNA damage are strongly diminished in the absence of XRCC1 (Fig. 2). A bona-fide explanation of this striking effect would be that even under non-stressed conditions more PARP1 molecules are bound to chromatin, due to potentially higher basal levels of DNA damage in *XRCC1* KO cells, and therefore, the pool of “free” PARP1 molecules available for recruitment to sites of laser-induced damage may be limited. What, however, contradicts this hypothesis are the findings by both Demin et al. and by us demonstrating that *XRCC1* KO cells do not display higher levels of strand breaks in the absence of genotoxic treatment (Fig. 5). We therefore hypothesize that DNA strand breaks are detected by PARP1, and its activation and subsequent automodification serve to recruit more PARP1 molecules. This view is supported by earlier experiments demonstrating that peak levels of PARP1 recruitment to sites of laser-induced DNA damage are diminished in case of PARP inhibitor treatment and in case of recruitment of the mono-ADP-ribosyl-transferase mutant PARP1/E988K (Rank et al. 2016). XRCC1 may then be required for the initiation of the next step, serving as a switch for the exchange of PARP1 molecules at the damage site, in line with results by Demin et al. (Demin et al. 2021). In the absence of XRCC1, such a repair cycle appears to be decelerated, leading to diminished recruitment and enhanced retention of PARP1 molecules and synthesis of shorter PAR molecules. Repair of the lesion might then be compromised, as access of downstream repair factors is impaired.

Looking at the interplay between PARP1 and XRCC1 from the opposite direction, we observed that recruitment of XRCC1 to the damage site was significantly reduced in the absence of PARP1 and could be rescued by PARP1 reconstitution (Fig. 2). These observations are consistent with the previously reported necessity for PARP1 activity for efficient XRCC1 recruitment and foci formation (Breslin et al. 2015; Okano et al. 2003), a role reported for both PARP1-depleted cells or cells treated with PARP inhibitor (El-Khamisy et al. 2003; Lan et al. 2004; Mortusewicz et al. 2007). Here, one should take into account that detected residual XRCC1 loading could be due to PARP2 activity, as redundant roles for PARP1 and PARP2 have already been identified in BER, as well as in the recruitment of endogenous XRCC1 in retinal pigment epithelial (RPE-1) cells (Hanzlikova et al. 2017a; Ronson et al. 2018). In support of this notion, previous experiments using potent PARP1/2 inhibitors complete abolished XRCC1 recruitment in different cell types (Campalans et al. 2013; Fischer et al. 2014). Another possibility for residual recruitment in *PARP1* KO cells could be PARP-independent recruitment of XRCC1. In this regard, Campalans et al. reported that XRCC1 recruitment to oxidative damage sites induced by micro-irradiation coupled to photosensitizers, or treatment with potassium bromate, was not affected by pharmacological PARP inhibition, which might in this case be directly mediated by 8-oxoguanine DNA glycosylase OGG1 (Campalans et al. 2013). Furthermore, the authors of that study showed that a functional BRCT1 domain of XRCC1 is vital for its recruitment to a single strand break site, but not to sites of oxidative damage, further illustrating different mechanisms for the recruitment of XRCC1 (Campalans et al. 2013).

Similar to the annexinV/PI cytotoxicity analysis of the present study using micromolar CPT concentrations (Figs. 3 and 4), Demin et al. revealed that deletion of *PARP1* in RPE-1 *XRCC1* KO cells nearly restored clonogenic survival rates upon MMS treatment to levels seen in *PARP1* KO cells. Furthermore, similar to our FADU DNA damage and repair analysis, which revealed a significant DNA repair defect of *XRCC1* KO cells upon H_2_O_2_ and CPT treatments that could at least partially be rescued by additional *PARP1* KO (Fig. 5), Demin et al. observed increased tail moments in Comet assay analysis in *XRCC1* KO cells upon MMS treatment—an effect that could be also rescued by additional PARP1 ablation (Demin et al. 2021). Taken together, both data sets provide strong evidence for the generality of the *PARP1*-KO-dependent rescue effect of the hypersensitivity of *XRCC1* KO cells towards several different genotoxic treatments and across different cell types. The fact that we did not observe an impairment in the repair of H_2_O_2_-induced lesions in *PARP1* KO cells, as observed before, is probably related to different treatment and analysis parameters, since in comparison to previous experiments, in the present study, we focused on rather late time points upon H_2_O_2_ treatment (up to 4 h after treatment). In general, it is well established that cells can better compensate effects of genetic deletion of PARP1 in comparison of cells treated with PARP inhibitors (Pandey and Black 2021). Thus, while PARP inhibitor treatment can lead to toxic PARP1 trapping at the site of the damage and therefore to manifestation of strand breaks, in case of genetic *PARP1* deletion, backup BER/SSBR repair pathways can still efficiently repair the damage, presumably because of redundancy with PARP2 during BER (Hanzlikova et al. 2017a; Ronson et al. 2018).

Interestingly, we did observe equal sensitivities of *PARP1* KO, *XRCC1* KO, and *PARP1/XRCC1* DKO cells towards nanomolar CPT concentrations with regards to G2 accumulation and clonogenic survival. In particular, the clonogenic survival data are in line with results reported by Demin et al.. While at first glance the annexin V/PI data (i.e., short term cytotoxicity after micromolar CPT treatment) and the clonogenic data (i.e., long-term “cytotoxicity” after nanomolar CPT treatment) may appear contradictory, in fact these data become conclusive when considering the different qualities of DNA damage induced by CPT: It can be hypothesized that in the short-term annexin V/PI cytotoxicity analysis after micromolar CPT treatment, SSBs are the predominant form of DNA damage, while in long-term clonogenic survival after nanomolar CPT treatment replicative stress including collapsed replication forks and DSBs may prevail. Thus, these data are in line with the hypothesis that PARP1 and XRCC1 cooperate in the repair of CPT-trapped Top1cc and associated SSBs, which leads in case of absence of XRCC1 to PARP1 trapping, continuous PARP1 activation, and NAD^+^ depletion. Instead, in case of CPT-induced replicative stress, resulting from collapsed replication forks and DSBs, which may predominate upon nanomolar CPT treatment, different mechanisms apply, during which the actions of PARP1 and XRCC1 appear to be epistatic.

A limitation of our study represents the use of the HeLa cell culture model. While HeLa cells represent a comprehensively studied human cell model in DNA damage and repair research (Forti 2016), of course, as a human cancer cell line, HeLa cells have their limitations with respect to the study of DNA repair mechanisms, because of gross genomic rearrangements, including dysregulation of p53 response mechanisms.

In conclusion, we demonstrate here that the functions of PARP1 and XRCC1 within the cellular genotoxic stress response are tightly interconnected and that the relationship between the two proteins is not only unidirectional, but reciprocal.

Data from the present study combined with the one by the Caldecott lab (Adamowicz et al. 2021; Demin et al. 2021) are consistent with and extend a model that has previously been proposed by Dianov and Hübscher (Dianov and Hubscher 2013). According to this model, one of the key functions of PARP1 during SSBR comprises the regulation of SSBR capacity and prevention of DSB formation by nuclease attack. In the light of this model, the available data is compatible with the following mechanistic scenario (Table 1): when SSBs form at levels at which the BER capacity is saturated, PARP1 can bind to excessive unprocessed SSBs, protecting them from uncontrolled attack by nucleases, and thereby preventing the formation of DSBs. This gives the BER machinery time to deal with the large number of lesions. The dynamic balance between PARP1 and PARG activity at the damage site leads to moderate steady state levels of automodified PARP1 and at the same time fosters the recruitment of XRCC1 and associated BER proteins as soon as they become available. Under those conditions efficient repair is possible in WT cells even under conditions of genotoxic stress. In *PARP1* KO cells, PARP1 is dispensable for the repair of SBBs at low levels, yet under conditions of genotoxic stress, this can increase the likelihood for the formation of DSBs, due to unprotected SSBs, leading to mutations and genomic instability. In contrast in *XRCC1* KO cells, BER is compromised even under conditions of low to moderate levels of DNA damage, however, other repair pathways may act as backup pathways, e.g., homologous recombination repair (HR), and translesion synthesis (TLS). Under conditions of high damage load in *XRCC1* KO cells, PARP1 still protects SSBs against nuclease attack, however, in this case initiation of BER is not possible due to the lack of XRCC1. Instead, trapping of PARP1 on SSBs and constant activation cycles lead to depletion of NAD^+^ and in consequence to a metabolic crisis. In contrast, in *XRCC1/PARP1* DKO cells, the BER defect of *XRCC1* KO cells can be rescued by genetic PARP1 ablation, since no toxic PARP1 trapping at SSBs can occur and NAD^+^ depletion and metabolic crisis can be prevented (Table 1). Future studies should show which mechanisms are at work in this case in order to enable repair in the absence of XRCC1 and PARP1. Good candidates are TLS and/or HR, but also XRCC1 and PARP1 independent BER activities may represent a possible scenario (Demin et al. 2021; Dianov and Hubscher 2013).

**Table 1:** Mechanistic summary reconciling results from the present and previously published studies (Adamowicz et al. 2021; Campalans et al. 2013; Demin et al. 2021; Dianov and Hubscher 2013). DSB indicates double strand break; HR, homologous recombination; SSB, single strand break; TLS, translesion synthesis

Genotype	Mechanistic and cellular consequences at physiological to moderate levels of SSBs	Mechanistic and cellular consequences at high levels of SSBs under conditions of genotoxic stress
WT	**Efficient BER possible without the involvement of PARP1**	**Efficient BER still possible with the involvement of PARP1** PARP1 protects SSBs that cannot be repaired immediately due to BER enzyme limitation, giving the BER machinery time to deal with the large number of lesions The dynamic balance between PARP1 and PARG activity leads to moderate steady-state levels of automodified PARP1 and at the same time recruitment of XRCC1 and associated BER proteins as soon as they become available to foster BER at damage sites
*PARP1* KO	**Efficient repair possible without the involvement of PARP1**	**BER still possible to some extent, e.g., via PARP1-independent access of XRCC1 to SSBs, or via HR, TLS** Loss of protection of SSB against nucleases, due to lack of PARP1, leading to an increased risk of DSB formation, mutations and genomic instability
*XRCC1* KO	**BER compromised** Other repair pathways may act as backup (e.g., HR, TLS)	**BER compromised** Other repair pathways may act as backup (e.g., HR, TLS) PARP1 protects SSB against nucleases, initially by “hovering” on them. But no completion of BER possible due to the lack of XRCC1 Trapping of PARP1 at SSBs and repeated PARylation cycles → **Depletion of NAD**^**+**^** / metabolic crisis**
*XRCC1* KO + PARP inhibitor	**BER compromised**	**BER compromised** PARP1 can protect SSBs against nucleases. However, since PARylation is pharmacologically blocked, PARP1 is trapped at lesions → **No depletion of NAD**^**+**^** / no metabolic crisis**
*PARP1/* *XRCC1* DKO	**BER working in an PARP1/XRCC1-independent manner** Other repair pathways may act as backup (e.g., HR, TLS)	**BER working in an PARP1/XRCC1-independent manner** Other repair pathways may act as backup (e.g., HR, TLS) Loss of protection of SSBs against nucleases, due to lack of PARP1, leading to an increased risk of DSB formation, mutations and genomic instability → **No depletion of NAD**^**+**^** / no metabolic crisis**

## Supplementary Information

Below is the link to the electronic supplementary material.

Supplementary file1 (PDF 249 KB)

## Data Availability

All data in this study are available from the corresponding author upon reasonable request.
