# Supplementary material for: PARP1 and XRCC1 exhibit a reciprocal relationship in genotoxic stress response
Source: Cell Biol Toxicol. 2022 Jul 1;39(1):345–64. doi: 10.1007/s10565-022-09739-9 (PMC10042965; doi:10.1007/s10565-022-09739-9)
Supplement: Supplementary file 1 — Supplementary file1 (PDF 249 KB) [file 10565_2022_9739_MOESM1_ESM.pdf]

SUPPLEMENTARY INFORMATION FOR

PARP1 and XRCC1 exhibit a reciprocal relationship in genotoxic stress response

Julia M Reber[1 §], Jovana Božić Petković[1 §], Michelle Lippmann[1], Marvin Mazzardo[1], Asisa Dilger[1], Rebecca Warmers[1], Alexander Bürkle[1 #], and Aswin Mangerich[1 # *]

[1] Molecular Toxicology Group, Department of Biology, University of Konstanz, 78457 Konstanz, Germany

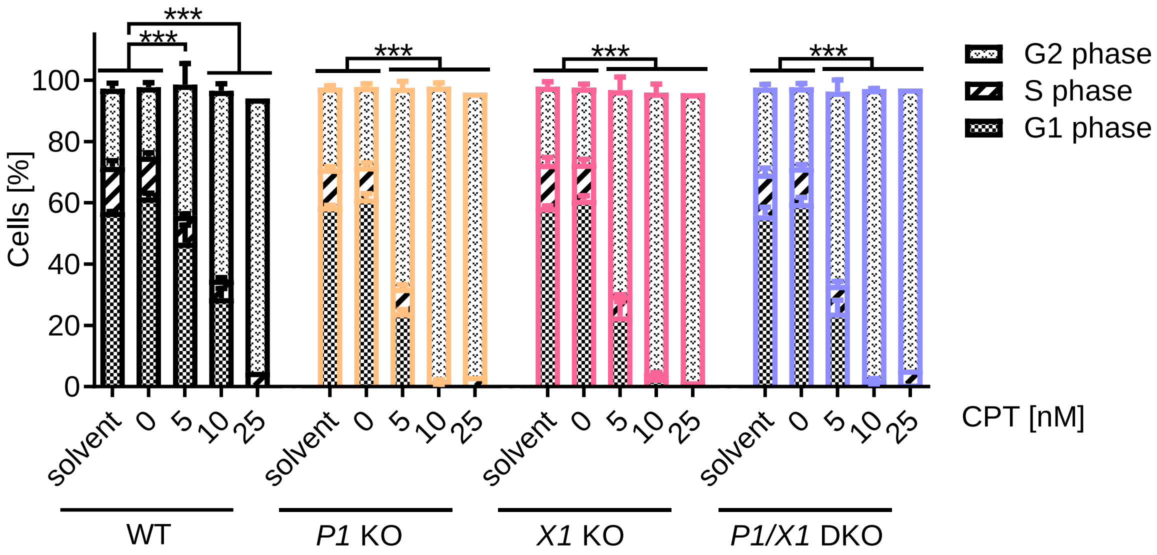

Supplementary Figure 1. Extended data of Figure 3 B, i.e., cell cycle analysis via PI staining and subsequent flow cytometry. Shown are the fractions of cells in G1, S or G2 phase after treatment with CPT for 42 h at concentrations as indicated. Means ± SEM of n = 3-5 independent experiments (n = 1 for treatment with 25 nM CPT). Statistical analysis was performed using two-way ANOVA testing with Tukey's post-test. Statistics are indicated only for cells in G2 phase.

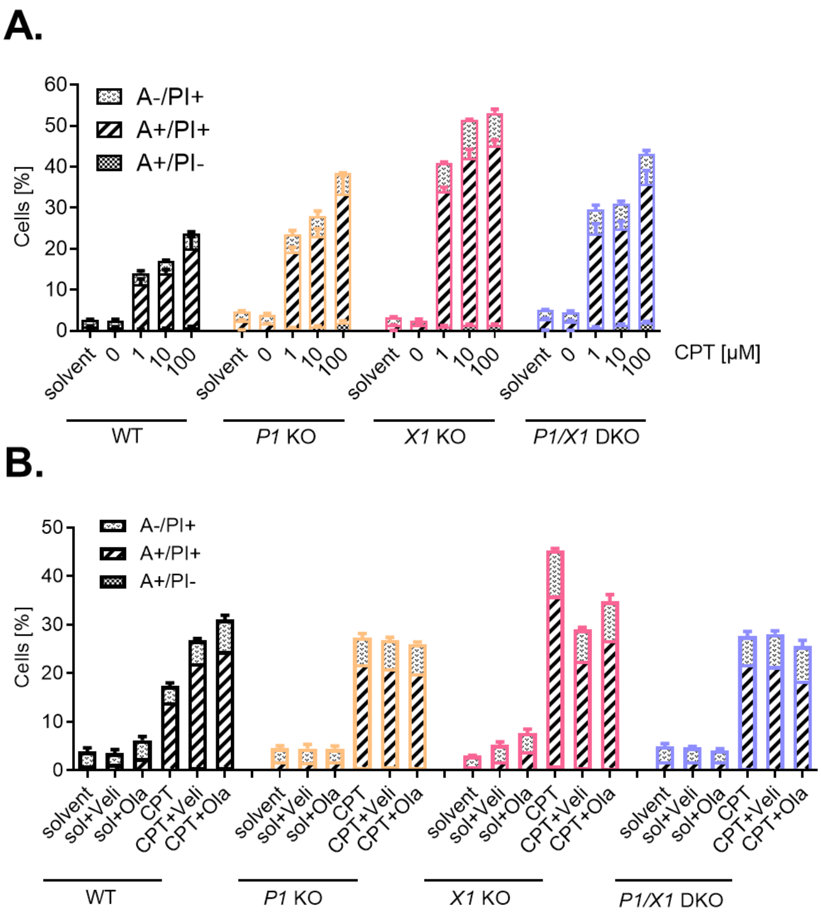

Supplementary Figure 2. Extended data of Figure 3 C and Figure 4 A, i.e., cytotoxicity analysis by Annexin V/PI staining and subsequent flow cytometric analysis. Fractions of Annexin V single positive (A+/PI-), popidium iodide single positive (A-/PI+) and Annexin V/PI double positive cells (A+/PI+) are shown. (**A**) HeLa WT and KO cell clones were treated with CPT for 42 h in concentrations as indicated. Means ± SEM of n = 3 independent experiments (extended data corresponding to **Figure 3C**). (**B**) HeLa WT and KO cell clones were treated with 10 µM CPT for 42 h ± 10 µM PARP inhibitor veliparib (Veli) or olaparib (Ola). Means ± SEM of n = 3 independent experiments (extended data corresponding to **Figure 4A**).
